# Respiratory-gated KES imaging of a rat model of acute lung injury at the Canadian Light Source

**DOI:** 10.1107/S160057751700193X

**Published:** 2017-03-21

**Authors:** P. Deman, S. Tan, G. Belev, N. Samadi, M. Martinson, D. Chapman, N. L. Ford

**Affiliations:** a Department of Oral Biological and Medical Sciences, The University of British Columbia, Vancouver, BC, Canada V6T1Z3; b Department of Physics and Astronomy, The University of British Columbia, Vancouver, BC, Canada V6T1Z3; c Canadian Light Source, Saskatoon, SK, Canada S7N2V3; d Department of Physics and Engineering Physics, University of Saskatchewan, Saskatoon, SK, Canada S7N5A9; e Division of Biomedical Engineering, and Department of Anatomy and Cell Biology, University of Saskatchewan, Saskatoon, SK, Canada S7N5A9

**Keywords:** *K*-edge subtraction, respiratory gated, *in vivo* imaging, contrast enhanced, lung injury

## Abstract

In this study, contrast-enhanced X-ray tomographic imaging for monitoring and quantifying respiratory disease in preclinical rodent models is proposed. A *K*-edge imaging method has been developed at the Canadian Light Source to very accurately obtain measurements of the concentration of iodinated contrast agent in the pulmonary vasculature and inhaled xenon in the airspaces of rats. To compare the iodine and xenon concentration maps, a scout projection image was acquired to define the region of interest within the thorax for imaging and to ensure the same locations were imaged in each *K*-edge subtraction (KES) acquisition. A method for triggering image acquisition based on the real-time measurements of respiration was also developed to obtain images during end expiration when the lungs are stationary, in contrast to other previously published studies that alter the respiration to accommodate the image acquisition. In this study, images were obtained in mechanically ventilated animals using physiological parameters at the iodine *K*-edge *in vivo* and at the xenon *K*-edge *post mortem* (but still under mechanical ventilation). The imaging techniques were performed in healthy Brown Norway rats and in age-matched littermates that had an induced lung injury to demonstrate feasibility of the imaging procedures and the ability to correlate the lung injury and the quantitative measurements of contrast agent concentrations between the two KES images. The respiratory-gated KES imaging protocol can be easily adapted to image during any respiratory phase and is feasible for imaging disease models with compromised lung function.

## Abstract 2

A *K*-edge subtraction imaging approach for respiratory-gated lung imaging using iodine and xenon contrast agents in a rodent model is presented.

## 1. Introduction   

Synchrotron computed tomography (CT) for *in vivo* imaging of small animals has some distinct advantages. Due to the monochromatic energies achieved, the measurement of the attenuation coefficients, the distinction of elements and their concentration is more accurate and sensitive to small variation than X-ray computed tomography (Bayat *et al.*, 2008 ▸; Adam *et al.*, 2009 ▸). Having a monochromatic source reduces the effects from beam hardening artefacts as well. Furthermore, the imaging geometry, with large source-to-object and object-to-detector distances results in a narrow beam profile with little scatter (Bayat *et al.*, 2008 ▸; Adam *et al.*, 2009 ▸). High-resolution imaging for investigations of cardiac and respiratory deformations in rodents has been described (Sera *et al.*, 2008 ▸). In the investigation by Sera *et al.* (2008 ▸), the rodents were placed onto a mechanical ventilator and image acquisition was performed during an iso-pressure breath-hold to eliminate artefacts due to physiological motion. The authors performed cross-sectional imaging based on X-ray attenuation and demonstrated, using an iodinated contrast agent, vessels and airways >0.25 mm in rats and >0.125 mm in mice.

In addition to absorption imaging, synchrotron imaging can also provide concentration measurements of contrast agents using *K*-edge subtraction (KES) techniques. In KES, images are obtained just above and below the *K*-edge of the contrast agent simultaneously and subtracted to yield an intensity map that is directly proportional to the concentration of the contrast agent in the different tissues (Adam *et al.*, 2009 ▸). Recently a spectral KES approach has been described, with improved quantization and sensitivity over traditional KES approaches (Zhu *et al.*, 2014 ▸). This method allows rapid changes in imaging energies (I, Xe and Ba *K*-edges) while requiring little realignment. Traditional KES has been demonstrated for measuring the regional concentration of xenon within the lung in rabbits in a histamine aerosol bronchial challenge model (Adam *et al.*, 2009 ▸; Monfraix *et al.*, 2005 ▸). Imaging was performed following introduction of 80% Xe in O_2_ and obtaining images during a 60 s apnea induced by pausing the ventilator at the end of inspiration and during functional residual capacity (Monfraix *et al.*, 2005 ▸). In another study, the radiation dose and image quality were studied for rabbits imaged with 70% Xe in O_2_ or 20% Xe in O_2_ (Strengell *et al.*, 2014 ▸). Following 21 breaths to reach steady state, the ventilator was paused for 3 s to obtain the images, with the authors concluding that low radiation doses acceptable for patient use were achievable with adequate image quality, but higher Xe concentrations were required which may lead to anaesthetic effects in the patient.

An alternative approach is temporal subtraction imaging (TSI), which subtracts two images, one pre-contrast and one post-contrast injection, to yield a distribution of the contrast agent similar to digital subtraction angiography. KES and TSI were compared by Suhonen *et al.* with good agreement on the calculated concentrations when the TSI images are registered prior to subtraction (Suhonen *et al.*, 2008 ▸). The TSI technique is more sensitive to tissue movements, as the two images could be obtained as much as 1 h apart, making registration a crucial step in this technique. Suhonen *et al.* integrated both methods to obtain simultaneous determination of Xe in the airspaces using KES and iodine in the vessels by TSI in mechanically ventilated rabbits (Suhonen *et al.*, 2008 ▸). Images were obtained during 4 s apnea by pausing the ventilator, and repeated to obtain the wash-in and wash-out curves of Xe and measure the ventilation. Similarly, images were obtained over 40 min at 2 min intervals during an infusion of a clinical CT contrast agent to obtain the wash-in and wash-out curves of iodine and measure perfusion.

In all of these KES studies, the animals were imaged during an apnea period initiated by the mechanical ventilator to eliminate motion. However, inducing apnea may alter the cardiovascular physiology, such as inducing vasoconstriction, and the Xe wash-in and wash-out curves will be affected by the ventilation parameters (Suhonen *et al.*, 2008 ▸). To assess a more realistic respiratory state, we have developed a method of acquiring the projection images during a predefined respiratory phase of a rat breathing at a physiologically relevant rate and tidal volume (Wiebe *et al.*, 2015 ▸). Our goal is to develop a method of imaging rodent respiratory structures and pulmonary vasculature together in rodent models of respiratory disease to collect more quantitative information about the disease state. Preliminary experiments have demonstrated that we can obtain respiratory-gated KES images that are synchronized with the real-time respiratory patterns of healthy rodents (Wiebe *et al.*, 2015 ▸). In the current study, we have used the respiratory-gated image acquisition to obtain KES images of iodine and xenon, present simultaneously in the animal, enabling characterization of the airspaces and vessels within the lungs of a lung-injury model and healthy controls. We anticipate that the KES synchrotron images could be used in the future to validate quantitative measurements of the iodinated contrast agents and inhaled xenon in micro-computed tomography studies.

In this study, we implemented respiratory-gated KES imaging to assess the distribution of an iodinated blood pool contrast agent in the pulmonary vessels *in vivo*, under mechanical ventilation at physiological parameters. We also performed KES imaging *post mortem* with a high xenon concentration to visualize the airspaces, keeping the mechanical ventilation at the same parameters as during the *in vivo* acquisition made less than 1 h before. Experiments were performed in a cohort of healthy rats and an age-matched lung injury model to demonstrate the feasibility of imaging a breathing animal using our respiratory-gating technique and the quantification of multiple contrast agents within the lung using KES images.

## 2. Methods   

### 2.1. Animal model   

All *in vivo* experiments were approved by the institutional animal care committee (University of British Columbia #A13-0290 and University of Saskatchewan #20130136), and were conducted on healthy male Brown Norway rats, approximately eight weeks old. Animals were group housed in a conventional animal facility, on a 12 h light–dark cycle and fed a standard laboratory diet. The animals were transported to the synchrotron facility approximately 24–48 h before their imaging sessions and allowed to acclimate in the temporary holding facility. The guidelines from UBC and USASK veterinary service were respected for all procedures and animal care.

To demonstrate the lung imaging techniques, we divided the animals into a control group (*n* = 4) and a lung injury group (*n* = 7). The control animals were imaged following the acclimation period with no additional experimental procedures. The lung injury group received an intratracheal instillation of lipopolysaccharide (LPS, 2.5 µg g^−1^ dissolved in saline) 24 h prior to imaging, which caused inflammation in the lungs and altered their respiratory health. Each animal was anaesthetized with an intraperitoneal injection of ketamine (75 mg kg^−1^) and medetomidine (0.5 mg kg^−1^) for the instillation. Once asleep, lidocaine was applied to the vocal cord and the animal was placed onto the tilted table, almost vertical to enable endotracheal intubation and LPS delivery. The animal was recovered and monitored every 4 h for respiratory complications for 24 h. Imaging was performed at 24 h post-LPS instillation.

### 2.2. Synchrotron facilities   

Imaging sessions took place at the Biomedical Imaging and Therapy (BMIT) bend-magnet beamline at the Canadian Light Source (CLS) using the spectral KES method described by Zhu *et al.* (2014 ▸). The beam configurations for imaging at xenon and iodine *K*-edges were firstly estimated by putting a tank of xenon and iodine in the beam. Three hundred projections per axial slice were taken. One image was composed of ten slices, taken from the bottom of the lung to the top (defined on the positioning radiography), equally spaced. The total acquisition time per *K*-edge was approximately 45 min, due to the gating (step and shoot acquisition). Each projection was acquired in the desired respiratory phase, then the stage was rotated to the next angular position and the system waited for the trigger signal before acquiring the next projection in the desired respiratory phase. The trigger signal was based on the respiration of the animal, as described in §2.4.

The detector (Hamamatsu flat panel sensor detector C9252DK-14) pixel size was 100 µm, and the height of the beam was around 80 µm at the waist (set at the center of the sample), leading to a resolution of approximately 100 µm × 100 µm × 100 µm. We used the flat panel detector in partial readout mode with the exposure time set to 6.8 ms per projection. However, at each position we had to acquire multiple images (3) to eliminate saturation caused by waiting for the respiratory trigger signal. The third image was used in the CT reconstruction and the others were discarded due to saturation. Thus, due to detector design each projection image actually takes three exposures to complete and the total imaging time was 3 × 6.8 ms = 20.4 ms per one projection image. The beam current was around 190 mA after refill and decreased to 170 mA just before refill, for an average beam current of approximately 180 mA.

### 2.3. Dose estimation   

The X-ray dose was measured free-in-air at the center of the field of view for each protocol to provide an estimate of the entrance dose to the animal. A calibrated thimble chamber system [dosimeter Accu-Dose Advance and thimble chamber 10×60-0.6-CT (0.6 cm^3^) from Radcal, Monrovia CA, USA] was used for dose measurements (measured three times and averaged).

### 2.4. *In vivo* imaging   

**Figure 1:**
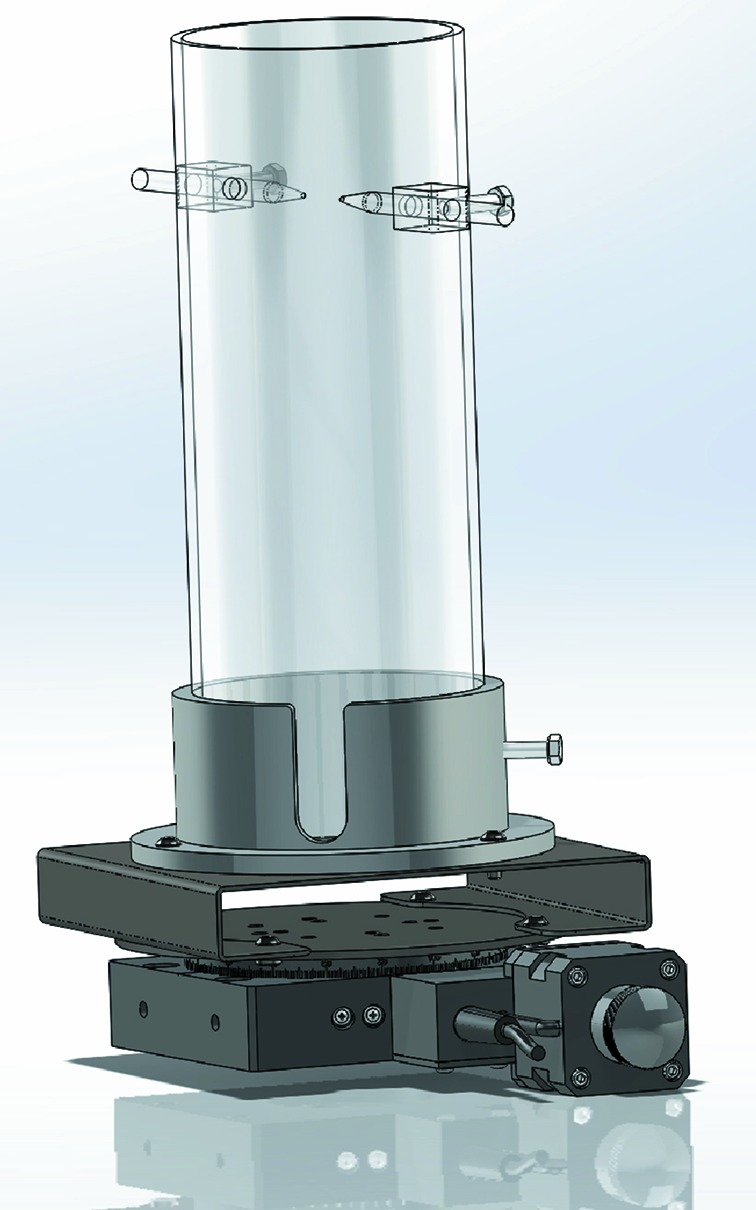
Custom-designed stereotaxic holder to position the rats vertically for the imaging sessions. The hole at the bottom allows access to the tail for the contrast agent injection *via* the tail vein catheter and manages the connectors for physiological monitoring. The holder is positioned on the rotation stage for acquisition of the projections for the CT reconstruction.

The rats were anaesthetized with inhaled isoflurane (5% in O_2_) in an induction chamber. Once anaesthetized, the rats were endotracheally intubated using a 16-gauge catheter and placed onto mechanical ventilation (Inspira, Harvard Apparatus Canada, St Laurent, QC, Canada). The ventilator was set to 82–83 breaths per minute with a tidal volume of 1.03–1.18 ml based on the individual body weights of the rats. Each rat was positioned in a custom-built stereotaxic holder, shown in Fig. 1 ▸. Anaesthesia was maintained throughout the imaging sessions with inhaled isoflurane (1.5–2% in O_2_). During the imaging session, the body temperature of the rats was kept at approximately 37°C using a heating lamp adjustable from the control room. The respiratory and cardiac frequencies, as well as body temperature were monitored throughout the imaging session using a physiological monitoring system [BioVet, Spin Systems, (QLD) Pty Ltd, Brisbane, Australia]. The cardiac frequency was measured using ECG pads on one rear and the two front paws, while the respiratory frequency was measured as a change in pressure in a pillow sandwiched between the rat’s diaphragm and the customized holder. The respiratory signal was used to trigger the image acquisition during end expiration to reduce artefacts due to respiratory motion.

The iodinated blood pool contrast agent eXIA 160XL (Binitio Biomedical, Inc., Ottawa, ON, Canada) was injected into the tail vein (160 mg I per kg body weight) *via* a catheter. Then the stereotaxic holder containing the anaesthetized rat was mounted vertically on the rotation stage. The first image was taken at the iodine *K*-edge. After iodine *K*-edge acquisition, we injected 0.15 ml per 100 g heparin into the tail vein catheter and euthanized the rat with an overdose of ketamine (135 mg kg^−1^) and medetomidine (0.75 mg kg^−1^). The ventilator was put on a closed circuit with a xenon gas source to ventilate the lungs with xenon, in order to consume less xenon which is very expensive. Rebreathing systems that enable reusing the xenon are not available for rodents, as the resistance within the filtration system is too great for a small rodent to accommodate. A second image was then recorded at the xenon *K*-edge, with a correction of the height of the sample and detector to adjust the geometry depending on the Bragg angle difference between the two *K*-edges in order to obtain axial slices at approximately the same position. As the iodinated contrast agent is a blood pool agent, which recirculates within the vascular system with no loss of contrast for over 1 h, the blood vessels contained iodine during imaging at the xenon *K*-edge. From the images, the iodine concentration and xenon concentration in the same areas within the lung were estimated.

### 2.5. Histological analysis   

Once the imaging session was completed, the animal was removed from the beamline. All monitoring equipment along with the endotracheal tube were removed and the lungs were surgically excised from the carcass. After the lungs were extracted, they were inflation fixed in formalin solution to preserve the tissue structure and architecture of the organ. To allow unfolding of the alveolar structures, formalin was instilled *post mortem* *via* the trachea by placing an IV bag with formalin at a 30 cm height above the highest point of the lungs. Once the lungs were extracted, they were immersed in formalin and shipped to our home institution for histological analysis.

Each lobe of the lung was sampled according to the systematic uniform random sampling procedure described by Hsia *et al.* (2010 ▸). A random cut was made near the top of the lung, and subsequent serial parallel sections of a constant thickness (0.4 cm) were made. The sections were flipped 90° such that the cut faces were visible. For each side of the lung, a section from the upper, middle and lower parts of the lung were taken to perform histological staining. In total, six sections were removed from each lung for further processing. The sectioned lungs were stored in cassettes and immersed in formalin. Subsequently, the sections were embedded in paraffin, sectioned to 10 µm using a microtome and then stained with hematoxylin and eosin (H&E). The sections were mounted onto glass slides and imaged with an Aperio slide scanning microscope (Leica Biosystems, Concord ON Canada) at 40× magnification.

### 2.6. Data analysis   

The synchrotron projections are reformatted line-by-line to produce a two-dimensional image for each angle and run through the SKES algorithm described previously (Zhu *et al.*, 2014 ▸) to subtract the two energies. The subtracted projections are then reconstructed using a filtered back-projection algorithm to make axial slices. The axial images were analyzed using *ImageJ* (National Institutes of Health, Bethesda, MD, USA). Regions of interest were drawn on the axial slices and separated into six groups (bottom, middle and top of the right and left lungs) corresponding to the approximate locations of the histological analysis. Measurements of the mean and standard deviations of the contrast agent concentrations in the iodine-enhanced and the xenon-enhanced regions were performed.

Mean and standard-deviation were calculated for the whole lung for LPS and control groups (over the ten slices). Moreover, as the LPS was not spread equally in the lungs, leading to areas of the lung affected by the LPS and others which were not, we subdivided all lung sections (from the LPS and control group) into two groups: lung regions with a percentage of inflammation measured in histology exceeding a threshold and lung regions with a percentage of inflammation measured in histology below the threshold. The threshold was chosen as the average of the percentage of inflammation in the control group plus five times the standard deviation of the percentage of inflammation in the control group. Then all sections of the lung in the LPS group that were not affected by the LPS will be clustered with the control group, avoiding averaging of LPS-affected lung sections with unaffected sections.

Histological images were analyzed using *ImageScope* (Leica Biosystems, Concord ON, Canada). To determine lung damage, the percentage of inflamed area of each lung section was quantified. A mask for the whole lung section was manually drawn on *ImageScope*. Regions of interest were chosen to include areas of inflammation characterized by an aggregation of white blood cells (such as basophils and neutrophils), which appear as a cluster of blue-stained nuclei in the H&E slides. The ratio of the inflamed area to the total area of the lung section provided the percentage of inflamed area. The number of regions of inflammation was also tabulated. The inflammation measured in the control animals will provide a background level of inflammation due to an un­related immune system response to bacteria, fungi, viruses and other irritants.

Statistical analysis of the histological sections was performed using *Prism* (version 6.0h, GraphPad Software Inc., La Jolla, CA, USA). Comparisons between the LPS group and the healthy controls were performed using two-tailed unpaired *t*-tests with Welch’s correction for unequal variances.

## 3. Results   

### 3.1. Dosimetry   

At the iodine *K*-edge the average dose was 0.255 mGy s^−1^ mA^−1^; at the xenon *K*-edge the average dose was 0.33 mGy s^−1^ mA^−1^. As we took 300 projections with an exposure time of 20.4 ms per projection, and assumed an averaged beam current of 180 mA, the total dose per scan is approximately 280 mGy and 364 mGy at the iodine and xenon *K*-edges, respectively. Due to detector design and issues with saturating the detector, we obtained three images at each angle but only the last image was used in the reconstruction, which led to a ‘useful dose’ of 93 mGy and 121 mGy, respectively.

### 3.2. Iodine and xenon concentration measurements   

**Figure 2:**
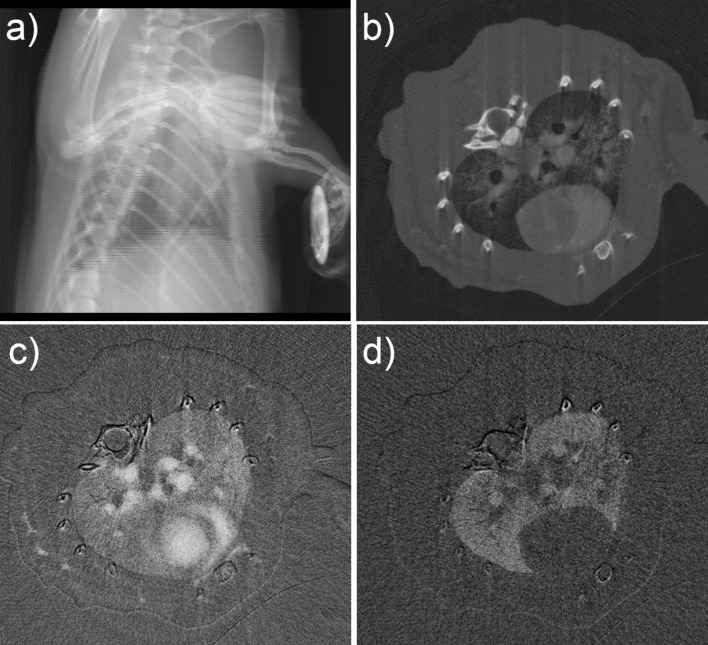
Synchrotron imaging data. (*a*) A projection image used to select the range for axial slices. (*b*) Tomographic reconstruction through the middle of the lungs and heart obtained during end expiration. (*c*) KES image of the iodine within the vasculature of the rat. (*d*) Corresponding KES image of the xenon in the airspaces of the lungs.

Fig. 2 ▸(*a*) shows the scout image for identifying the desired region of interest. Fig. 2 ▸(*b*) shows the CT reconstruction of a slice through the middle of the lungs, with the corresponding KES images for iodine and xenon shown in Figs. 2 ▸(*c*) and 2(*d*), respectively. No significant differences were found between the LPS and the control group for the iodine and the xenon concentrations, as well as between the group of inflamed lung section and not inflamed lung section as shown in Table 1 ▸. However, separating the regions that were unaffected by the LPS instillation and grouping them with the controls (Table 1 ▸, lung section < threshold) lead to differences in the % inflammation and the xenon concentrations compared with the affected regions (Table 1 ▸, lung section > threshold).

### 3.3. Histological analysis   

**Figure 3:**
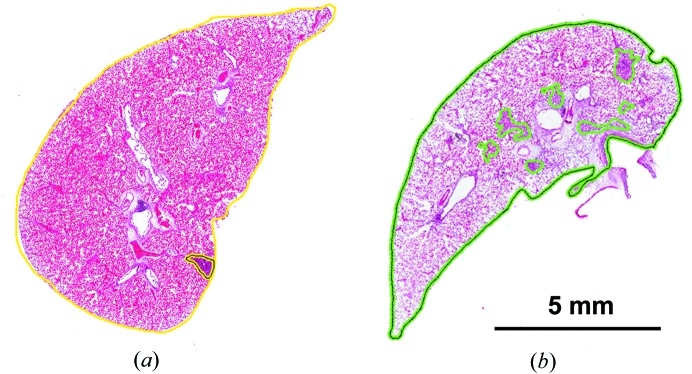
Representative H&E slides for (*a*) a control rat and (*b*) a rat instilled with LPS. Areas of inflammation are outlined within the lung tissue. The % inflammation for each slide is calculated as the total area of inflamed regions divided by the entire tissue area.

Sample slides from the LPS and the control animals are shown in Fig. 3 ▸. The outer boundary on each slide delineates the full area of the lung section. The inner contours outline areas identified as inflammation and the total area of the inflamed regions was expressed as a percentage of the entire lung section. The large voids represent the airways passing through the section.

The inflammation is quantified in Table 2 ▸ as a percentage of the entire lung section. To create this table, we pooled the measurements of the corresponding slides for each animal and then calculated the mean and the standard deviations for each group. For each of the regions listed in Table 2 ▸, the differences between the LPS and the control animals were statistically significant (*p* < 0.05). For Table 3 ▸, the measurements were compared directly for each of the six locations sampled. All of the measurements were statistically significant (*p* < 0.05) except for the middle region of the left lobe. We believe that this exception is due to the variability in introducing the LPS into the lungs, as controlling the aerosolized particles once instilled is not feasible, and due to the small number of samples obtained from each animal.

The number of inflammation sites is given in Table 4 ▸. Most of the slides show no significant differences in the number of inflammation sites, despite the LPS animals exhibiting an increased area of inflammation in all regions. The LPS animals had a large variation in the measured inflammation in the different lung regions, and the tissue was not uniformly affected by the LPS challenge, which resulted in *p*-values that were not statistically significant. Only the lower-right and the middle left lobes showed a significantly increased number of inflamed regions in the LPS animals compared with controls (*p* < 0.05).

## 4. Discussion   

This study is one of the first *in vivo* lung imaging experiments at the CLS using the KES method. We developed a scout projection image to improve the positioning of the animal and define which slices of the lung will be acquired. The scout view enabled accurate positioning for imaging the whole lung at the same positions for the iodine *K*-edge and the xenon *K*-edge. Control for anaesthesia and a heating lamp outside of the hutch allowed for adjustment during the long scan period.

Image acquisitions were gated to obtain projection images during end expiration, which is the period between breaths when the motion of the lungs is minimized. Gating the images reduced the motion due to respiration of the animal, and ensured that structures were not moving out of the axial plane between projections. The images obtained had good definition of the structures, with low X-ray doses of around 100 mGy, and little blurring due to motion. Gating also enabled good correlation between the iodine and xenon KES image acquisitions, such that the same locations in the lungs were imaged. Our gating technique is different from previously published studies as the images were obtained in breathing animals, whereas others have paused the ventilator to image during apnea (Monfraix *et al.*, 2005 ▸; Strengell *et al.*, 2014 ▸; Suhonen *et al.*, 2008 ▸). We expect that our technique will provide more physiologically relevant conditions to obtain quantitative measurements of air content in the lungs, blood volume, *etc*.

We had to euthanize the rats for the xenon imaging because we do not yet have a rebreathing system for small animals. Indeed, most of the rebreathing systems, which would allow us to recycle the xenon for *in vivo* imaging, use filters (for example, soda lime) and the gas will pass through only with a certain pressure. This minimum pressure is too high for rats on most of the available systems, which need animals at least as big as rabbits. To obtain true *in vivo* results, a custom-built xenon rebreathing apparatus suitable for rodents will be necessary.

From the images obtained, the concentrations of iodine and xenon within the thorax were calculated. Because the images were gated to end expiration, the lungs and airways were in the same respiratory phase and the images could be correlated between the two KES acquisitions. For the lung injury model, we found no differences in the measured xenon and iodine concentrations compared with control animals; however, for the functional airways, it may have been better to image just after peak inspiration to capture the lungs while fully inflated.

Our histological analysis showed that inflammation was present in the lung injury model, although the LPS instillation did not uniformly affect the lung tissues. Other markers of lung damage that may be measurable in the KES images include changes in the major airways and vascular remodeling. In our LPS model we expected that the xenon concentration may be affected, but we did not expect that the iodine concentration (representing the blood vessel volume) would be modified in this 24 h model of early and acute lung injury. To account for the non-uniform distribution of the LPS within the lungs, we separated the lung sections based on the presence of inflammation (measured in histology) so that the regions of the lungs unaffected by LPS (low inflammation) were grouped with the control animals. In this way, the differences between groups were more apparent, with increased xenon concentration in the LPS-affected regions, corresponding to increased air in the airspaces and inflammation, but with no changes in the vasculature. This study demonstrates that KES imaging with xenon and iodine provides quantitative measurements of contrast agent concentration that can be obtained during a prescribed respiratory phase in healthy and respiratory-compromised animals. Furthermore, both contrast agents were accurately identified with KES even when they were both present simultaneously.

Images obtained in this study were at 100 µm isotropic resolution. This resolution does not allow direct estimation of alveolar inflation as reported by Chang *et al.* (2015 ▸) which is a better indicator of alveolar damage than iodine and xenon concentration maps. But the area examinable by these techniques seems very small, which is a huge limitation, particularly for disease models that do not uniformly affect the lung tissues such as our LPS challenge. In addition, the technique described by Chang *et al.* does not allow for measurements of the ventilation globally within the lungs.

Improvements to the experimental setup for the future include increasing the number of slices obtained through the lung and performing all imaging *in vivo.* A rebreathing system to allow xenon imaging on live animals would be a great improvement of our experiment. Part of the displacement of the lung tissues that we observed between the iodine imaging and xenon imaging session is due to the fact that we have to euthanize the rat to put the animal on a closed circuit, saving xenon, which is very expensive. Following euthanasia, the lung tissues collapsed, leading to some minor mis-registration between our images. However, we do not expect any physiological modifications to the airspaces in the lung compared with the *in vivo* situation. A custom-built rebreathing system suitable for the low tidal volumes of rodents will have to be designed and tested for use with inhaled xenon contrast agents.

One of the main limitations in the KES technique used in our experiments is the impossibility of performing more than one slice per rotation. So it will not be possible to acquire a full lung in an acceptable acquisition time even if we improve the step-and-shoot method. For full-lung imaging, a laboratory-based micro-CT method, such as that described by Ashton *et al.* (2014 ▸), is better. But distinguishing between xenon and iodine, with really close *K*-edge energies, may not be possible using micro-CT, and these are the two most common contrast agents for vascular and airways imaging, respectively, and the only agents available clinically. We propose that synchrotron imaging will be valuable for calibrating and validating the micro-CT imaging techniques to enable quantitative measurements of contrast agent uptake within the different tissues.

## 5. Conclusions   

In this study, we have developed a technique of obtaining KES images for two different contrast agents in a specified respiratory phase of a rat lung *in vivo*. To ensure registration between the iodine and xenon KES images, we improved the positioning procedure to ensure the slices covered the same region of the lung and acquired the images during the period of least motion corresponding to end expiration. The technique was demonstrated in healthy rats and in a lung-injury model to demonstrate feasibility of the imaging techniques and the quantitative measurements that can be obtained.

## Figures and Tables

**Table 1:** Tabulated inflammation measured in histology and concentration values (g ml^−1^) measured in the synchrotron images of healthy controls and the lung injury model Values are grouped based on treatment group (LPS *versus* control) and regrouped based on the inflammation locations within the lung (below threshold = low inflammation; above threshold = inflammation induced by LPS). Values shown are mean ± standard deviation.

Group	LPS	Control	Lung section < threshold	Lung section > threshold
Inflammation (%)	5.1 ± 5.3	0.45 ± 0.88	1.29 ± 1.29	9.1 ± 6.0
Iodine concentration	0.0043 ±0.0025	0.0053 ± 0.0018	0.0046 ± 0.0020	0.0046 ± 0.0030
Xenon concentration	0.009 ± 0.003	0.011 ±0.003	0.0100 ± 0.0037	0.0093 ± 0.0021

**Table 2:** Average inflammation observed in the H&E slides separated into regions expressed as a percentage of the total area Values are the mean and standard deviations, and the *p*-values are from unpaired *t*-tests with Welch’s correction for uneven variances. All comparisons were significantly different between the LPS and control groups (*p*<0.05).

	LPS group (*n* = 7)	Control group (*n* = 4)	*p*-value
Whole lung (six slides per rat)	4.6 ± 1.6	0.4 ± 0.7	0.0003
Left lung (three slides per rat)	5.3 ± 2.7	0.5 ± 0.7	0.0026
Right lung (three slides per rat)	3.8 ± 1.3	0.4 ± 0.7	0.0003
Upper region (two slides per rat)	4.4 ± 1.9	0.4 ± 0.7	0.0009
Middle region (two slides per rat)	5.9 ± 5.5	0.6 ± 0.9	0.0440
Lower region (two slides per rat)	3.5 ± 1.7	0.4 ± 0.5	0.0018

**Table 3:** Percentage inflammation observed in the H&E slides in each of the six sampling regions expressed as a percentage of the total area Values are the mean and standard deviations, and the *p*-values are from unpaired *t*-tests with Welch’s correction for uneven variances. All comparisons were significantly different between the LPS and control groups (*p*<0.05) except the middle-left region. (ns) = no significant differences.

	LPS group (*n* = 7)	Control group (*n* = 4)	*p*-value
Upper right	3.8 ± 1.4	0.7 ± 1.4	0.0107
Upper left	4.9 ± 3.9	0.1 ± 0.1	0.0172
Middle right	4.2 ± 3.9	0.3 ± 0.2	0.0363
Middle left	7.5 ± 8.5	0.9 ± 1.8	0.0871 (ns)
Lower right	3.5 ± 1.8	0.3 ± 0.5	0.0027
Lower left	3.6 ± 2.3	0.4 ± 0.5	0.0098

**Table 4:** Number of inflammation sites observed in the H&E slides Values are the mean and standard deviations, and the *p*-values are from unpaired *t*-tests with Welch’s correction for uneven variances. All comparisons showed no significant differences (ns) between the LPS and control groups except for the lower-right and middle-left regions.

	LPS group (*n* = 7)	Control group (*n* = 4)	*p*-value
Upper right	4.3 ± 2.3	3.3 ± 5.9	0.7535 (ns)
Upper left	4.4 ± 4.0	0.8 ± 1.0	0.0526 (ns)
Middle right	8.9 ± 6.0	3.0 ± 2.7	0.0540 (ns)
Middle left	8.9 ± 7.1	1.3 ± 1.9	0.0297
Lower right	6.7 ± 5.6	1.0 ± 1.4	0.0369
Lower left	7.7 ± 3.4	3.0 ± 5.4	0.1808 (ns)
